# eHealth and Telemedicine Applications in Climate Change Mitigation: A Comprehensive Analysis for the Health Information Exchange, Care Collaboration, and Patient Engagement domains

**DOI:** 10.1055/s-0046-1816610

**Published:** 2026-11-03

**Authors:** Thomas Schabetsberger, Patrick Mangesius, Alexander Hörbst

**Affiliations:** 1 Siemens Healthcare DiagnosticsViennaAustria; 2 Siemens Healthcare Diagnostics, Vienna, Austria; Scientific Society for Health Information Systems (WIGIS)MilsAustria

**Keywords:** Digital Health, Environmental Sustainability, Carbon Footprint, Health Information Systems, Telemedicine, Climate Protection

## Abstract

**Background:**

Healthcare systems account for approximately 4.4 to 4.6% of global greenhouse gas emissions. This represents a critical contradiction, as the very sector dedicated to protecting human health is contributing to environmental degradation, which in turn threatens health. Digital health technologies have the potential to provide a solution, as they enable ways to maintain optimal healthcare while protecting the environment.

**Objective:**

The goal was to investigate how digital health technologies contribute to environmental sustainability through three primary mechanisms: Health Information Exchange systems, Care Collaboration, and Patient Engagement. This analysis evaluates empirical evidence for CO2 emissions reduction and considers the European Health Data Space initiative, as well as applications and experiences from different geographic regions.

**Methods:**

We conducted a comprehensive literature review focusing on peer-reviewed studies from 2012 to 2025 that quantified the environmental impacts of implementing digital health. The selection criteria prioritized studies with transparent methodological approaches and measurable environmental outcomes. Regional case studies were included to understand different implementation strategies and their environmental consequences.

**Results:**

The analysis of large-scale systematic reviews covering more than 68 million digital health consultations shows significant benefits for the environment. CO2 savings per virtual consultation range from 0.70 to 372 kg CO2 equivalent, with differences primarily attributable to geographical factors, transport structures, and healthcare models. Avoiding unnecessary duplicate tests and reducing waste for paper and medications also make significant contributions. At the same time, the energy consumption of the technological infrastructure accounts for less than 1% of the emissions avoided through reduced travel.

**Conclusions:**

Digital health technologies show considerable potential for reducing the environmental footprint of healthcare while maintaining or even improving clinical outcomes. To support this, it is necessary to establish standardized measurement tools, create policy frameworks, apply standardized interoperability approaches, and carefully consider equal treatment in implementation.

## 1. Introduction

Optimal healthcare delivery and environmental sustainability represent one of the most compelling challenges facing our current society. While healthcare organizations are committed to protecting and improving human health, this sector surprisingly contributes to environmental degradation to an extent that cannot be neglected. This ultimately threatens the populations it serves [ 1 ]. This contradiction leads to an increasing attention to sustainable healthcare practices that can maintain or even enhance clinical excellence while reducing its negative environmental impact.

However, healthcare’s environmental footprint is varying across regions and healthcare system structures. High-income countries show proportionally higher healthcare emissions, with the United States leading at the way with 8.5% of its national emissions, followed by Australia with 7.0% and Germany with 5.2% [ 2 , 3 ]. The comparison of national carbon footprint proportions caused by healthcare between those countries is outlined in Figure 1 , whereas healthcare emissions include facility operations, medical equipment manufacturing, pharmaceutical production, waste management, and transportation of patients and staff [ 4 ].

**Figure 1:**
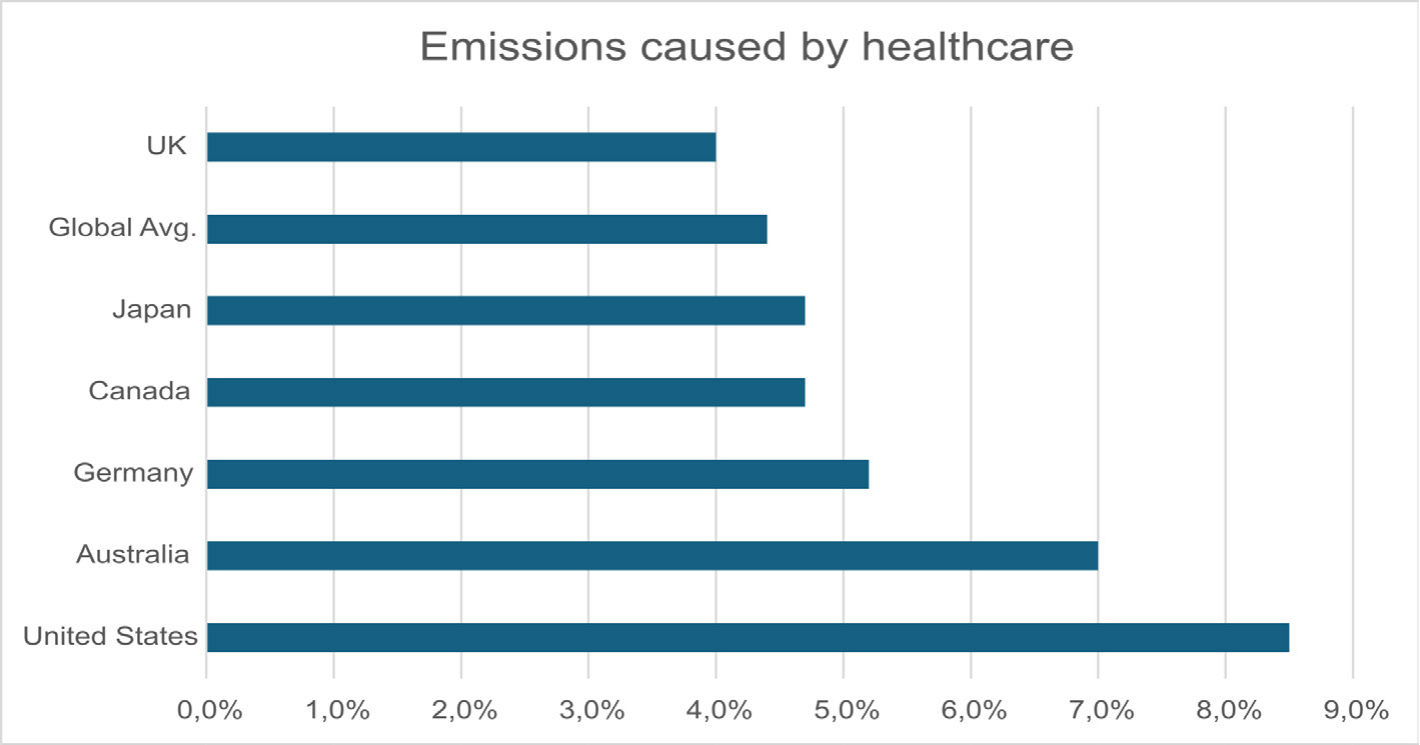
Percentage (%) of carbon dioxide emissions caused by the healthcare system in relation to the country’s total emissions.

The emergence of digital health technologies coincides with the growing awareness of healthcare’s environmental impact, creating opportunities to address both health-related and sustainability objectives at the same time. The COVID-19 pandemic accelerated digital transformation globally, providing large scale evidence in virtual care delivery and revealing challenges and possibilities for sustainable transformation [ 5 ].

The adoption, implementation, and use of eHealth and telemedicine applications in regions worldwide is influenced by a variety of factors. According to the authors, the lowest common denominator across regions worldwide can be seen as: (A) the availability and thus the exchange of health data, (B) the possibility of interdisciplinary and trans-institutional cooperation between healthcare professionals, and (C) the involvement and empowerment of patients. This paper deliberately avoids considering other exciting technological innovations, as their regional availability and thus their comparability varies.

Therefore, this work aims to concentrate on three principal domains where digital health technologies intersect with environmental sustainability:

- Health Information Exchange (HIE): which amongst others reduces paper consumption, postal services, telecommunication efforts and redundant procedures;
- Care Collaboration (CC): which e.g. impacts travel-related emissions, clinical errors or the duration of treatment;
- Patient Engagement (PE): to e.g. promote self-management or to decrease the healthcare facility utilization frequency.

The comparison of Europe, North America, Latin America, and Asia-Pacific regions, allows for the exploration on different approaches to digital health deployment and its influence on health-related and environmental goals. Overall, the African continent is currently responsible for a relatively small proportion of the CO2 footprint. Today, healthcare is still largely focusing on providing basic medical care, where SMS and telephone calls are used as technical tools according to [ 6 ]. Therefore, Africa was not analyzed within this study.

The aim of this paper is to determine, based on existing studies and evidence, whether and to what extent HIE, CC and PE can contribute to reducing the share of the carbon footprint caused by healthcare.

This paper provides a summary of the current state of knowledge on climate protection through eHealth and telemedicine applications, and presents another set of arguments for countries, healthcare institutions, and technology providers introducing these technologies.

## 2. Methodology

### 2.1 General Search Strategy

A comprehensive literature review has been combined with regional case study analysis to understand the quantitative environmental impacts and the qualitative implementation factors.

The databases that have been used are the following:

- MEDLINE, through PubMed (database for biomedical literature);
- Web of Science Core Collection (interdisciplinary);
- Scopus (scientific literature);
- EMBASE (European medical literature).

As detailed below, the search terms and keywords consisted of a combination of Medical Subject Headings (MeSH) terms and free-text keywords, limited to the English language.

#### Cluster 1: Digital Health Technologies

- eHealth” OR “electronic health record” OR “digital health” OR “Health informatics”
- telemedicine” OR “telehealth” OR “remote care” OR “virtual care” OR “care collaboration”
- health information exchange” OR “HIE”
- mobile health” OR “mhealth” OR “digital therapeutics”
- remote patient monitoring” OR “RPM” OR “connected health”
- patient portal” OR “personal health record” OR “phr”

#### Cluster 2: Environmental impact

- climate change” OR “global warming” OR “greenhouse gas”
- carbon footprint OR “carbon emission” OR “CO2 equivalent”
- sustainability” OR “environmental impact”
- green healthcare” OR “sustainable healthcare”
- energy consumption” OR “resource utilization”

#### Cluster 3: Healthcare domains

- Health information exchange” OR “care coordination” OR “care collaboration”
- patient engagement”
- healthcare delivery” OR “health service”
- primary care” OR “specialist care” OR “chronic disease management”

#### Regional dimensions

- Europe” OR “European Union” OR “EU” OR “European Health Union” OR “European Health Data Space”
- North America” OR “United States” OR “USA” or “Canada”
- Latin America” OR “South America” OR “Mexico” OR “Brasilia” OR “Columbia” OR “Chile” OR “Argentina” OR “Peru”
- Asia” OR “China” OR “Japan”

The search strategy was conducted by using a combination of Cluster 1-3 terms combined with the AND Boolean operator.

#### Inclusion criteria were:

- Peer-reviewed research, systematic reviews and meta-analyses;
- Conference proceedings;
- Technical reports;
- Studies addressing environmental impact;
- Assessments of carbon footprint, energy consumption or sustainability;
- Implementation and project articles with focus in HIE, CC or PE.

We specifically looked for studies published between January 2012 and May 2025, which use environmental impact measurements and carbon footprint calculations. Titles and abstracts of the identified articles were screened. To maintain an analytical focus and ensure that only the highest quality evidence was included, we limited our reference list to approx. 30 carefully selected sources and used the Claude AI service 1 .

The three areas - HIE, CC, and PE - represent key components of modern, integrated care from a practice-based perspective, as such they are also part of many healthcare companies’ product lines. Together with the respective regions, they were selected for comparison and enriched with prior practical experiences from our industry implementation projects, allowing for a holistic assessment.

### 2.2 Prisma Flowchart

The following Prisma chart in Figure 2 outlines the implementation of the search strategy.

**Figure 2:**
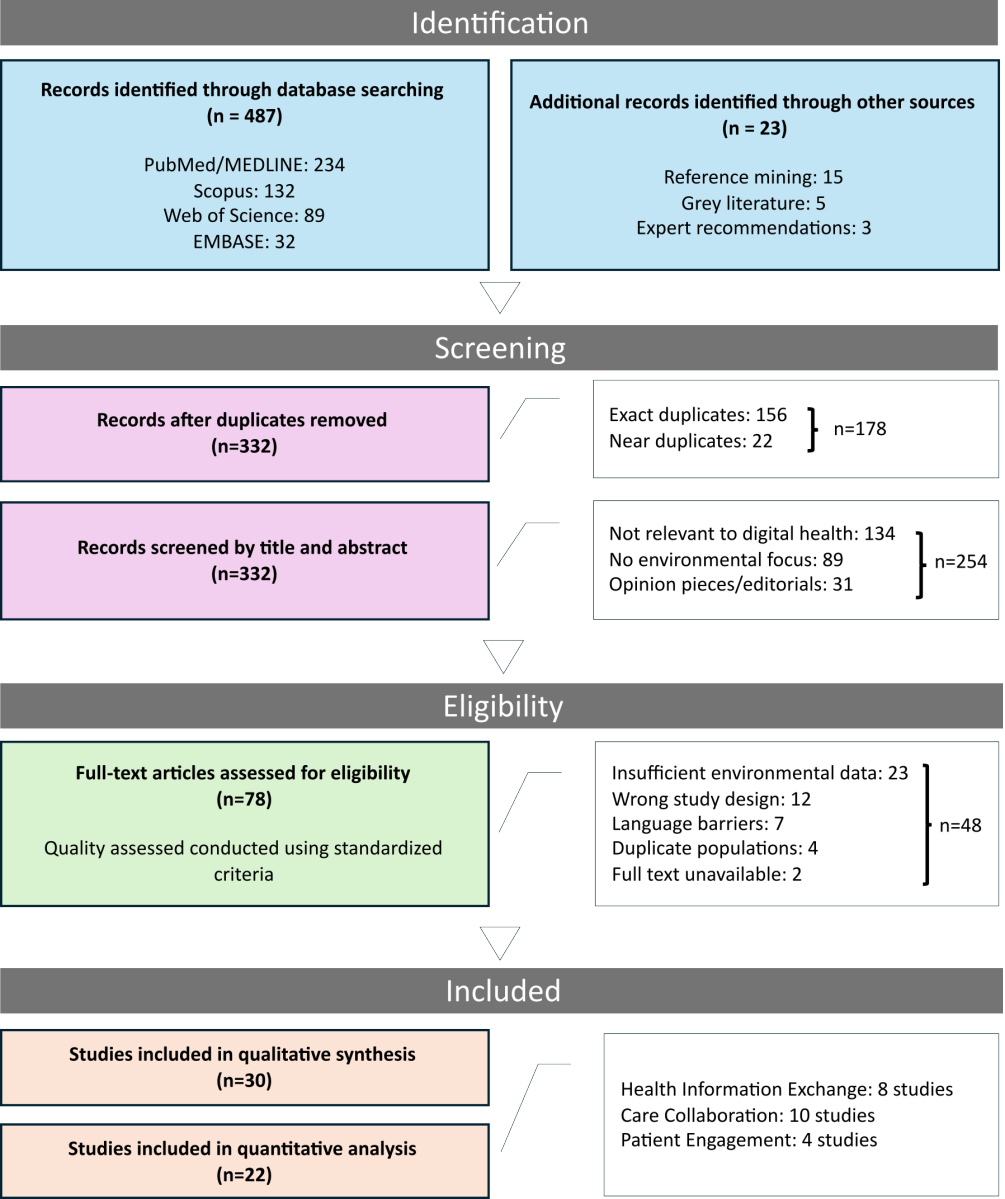
The Prisma flowchart explains the implementation of the search strategy and shows the interim results during the process. The final 30 studies in qualitative synthesis represent the core studies that form the foundation of the review, while 22 of them show measurable environmental impact data.

## 3. Current Evidence for Environmental Impact

### 3.1 Systematic Review Findings

The evidence for digital health environmental benefits results from comprehensive systematic review articles that have analyzed multiple studies of different healthcare contexts. These articles consistently demonstrate substantial carbon emission reductions associated with virtual care delivery, however, whereby the levels of reduction differ considerably from each other [ 7 , 8 ].

Purohit and colleagues conducted one of the most influential systematic reviews in this area [ 9 ]. They analyzed studies comparing the carbon emissions of traditional in-person/physical consultations with virtual alternatives. Their analysis found that the analyzed studies unanimously concluded that the use of telemedicine reduces the carbon footprint of healthcare, primarily by reducing transportation emissions. The magnitude of the benefits varied widely, ranging from 0.70 to 372 kg CO2 equivalent per consultation, due to the different contexts and measurement approaches used in the studies (see Figure 3 ).

**Figure 3:**
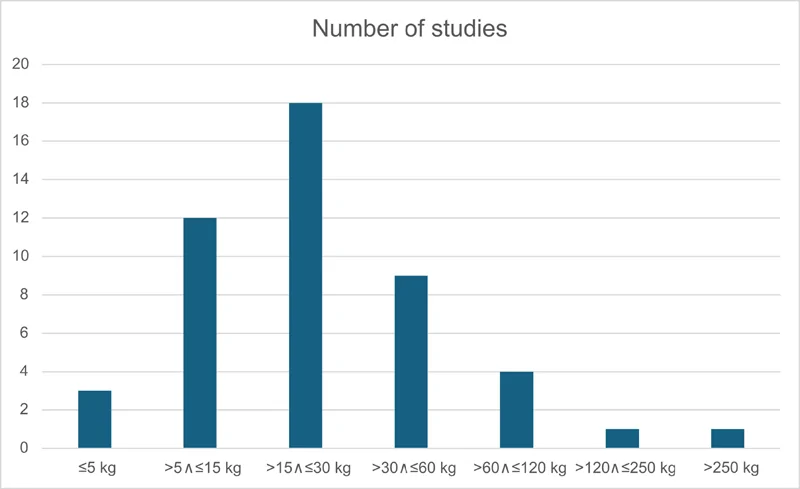
The comparison of different studies shows considerable deviations in the CO2 reductions described [ 4 , 5 ]. These deviations result from different methodological approaches and geographical differences.

This figure becomes even clearer when compared with global beef consumption. Assuming 14 kg of CO2 per kg of beef, a single consultation (372 kg of CO2) is equivalent to 26 kg of beef. Or, based on global annual consumption of 9 kg per person, the annual beef consumption of 2.9 people.

More recent analyses have further refined the understanding of the factors influencing the magnitude of the environmental impact. In their work, Van der Zee and colleagues provided updated data that also confirmed the minimal energy consumption of digital health technologies compared to avoided transportation emissions [ 10 ]. Their analysis found that the technological infrastructure typically consumes 0.04-0.052 kg CO2 equivalent per consultation hour, which is less than 1% of the total emissions reduction.

### 3.2 Methodological Considerations and Need for Standardization

Despite consistent findings regarding environmental benefits, the field faces significant challenges in terms of methodological standardization and comprehensive impact assessment. Lokmic-Tomkins and colleagues conducted a comprehensive review study that identified significant gaps in existing research approaches and noted the lack of standardized methods for assessing the environmental impacts of digital health [ 11 ].

This methodological diversity complicates comparative analyses and limits the ability to make informed predictions about environmental benefits in new application contexts. The complexity of environmental assessment of digital health services goes beyond simple calculations of traffic reduction and includes more far-reaching impacts at the system level. Modern health information systems influence examination or testing patterns, drug prescription behavior, the efficiency of care coordination, and resource use in various areas of healthcare.

A comprehensive environmental assessment requires “holistic approaches” that take these interrelated impacts into account rather than focusing exclusively on the direct impacts of transportation [ 11 ].

## 4. Health Information Exchange

### 4.1 Digital Infrastructure Environmental Benefits

HIE systems create environmental benefits through multiple mechanisms that extend beyond the obvious reduction in paper consumption. While the transition from paper-based to electronic health records reduces substantial printing and storage and (often postal) sending requirements, the more significant environmental benefits mostly arise from better care coordination and reduced redundancy in healthcare delivery.

Modern HIE systems enable healthcare providers to access comprehensive patient information across institutional boundaries, reducing the likelihood of duplicate testing or examinations, redundant procedures, and unnecessary specialist referrals. These efficiency gains lead directly into environmental benefits through reduced energy consumption of diagnostic equipment, reduced pharmaceutical waste, and better facility utilization [ 12 ].

The implementation of standardized interoperable technologies further amplifies these positive effects by reducing the overhead required for integration, data harmonization, and interface translation. When health systems can efficiently share information using well-established common standards and protocols, the overall energy consumption for data processing and storage is minimized while care quality is improved.

Another development which has influenced the whole information technology sector and subsequently the healthcare sector in recent years are cloud technologies, virtualization, and the establishment of computational platforms, together with available standards and best practices in healthcare that support the use of these underlying technological layers. There are several studies, e.g. [ 13 , 14 ], that show a positive impact on energy consumption and report savings of up to 30% using power-aware algorithms [ 13 ] in cloud-computing. Examples are outlined in Table 1 .

**Table 1:** Four examples of HIE functions that show environmental benefits, with estimated impacts and real-world implementation examples.

**HIE Function**	**Mechanism**	**Estimated Impact**	**Project Example**
Electronic documentation and sharing of results	Reduction of paper	substantial reduction of paper usage	NHS (UK) [ 12 ]
Lab result or image sharing	Reduction of duplicate examinations or tests	15-30% reduction	Regional health networks [ 9 ]
Electronic prescriptions	Prescription efficiency	Reduced medication waste	E-prescription systems [ 15 ]
Care coordination	Optimized resource utilization	Efficiency improvement	Integrated delivery networks [ 12 ]

### 4.2 European Health Data Space: A framework for sustainability in healthcare

The European Health Data Space (EHDS) is the technical concept for establishing a European Health Region and, among other benefits, is perhaps the most ambitious attempt to create an ecologically sustainable health information infrastructure on a near-continental scale. It was officially launched in March 2025 and sets binding interoperability requirements and data exchange protocols for all 27 member states of the European Union [ 16 ].

From an environmental perspective, the EHDS provides various opportunities for reducing emissions. Standardizing health data formats across borders avoids redundant data collection when patients are treated in different countries. The (new) possibility of secondary data use enables population-level health studies in principle and dramatically facilitates data collection for scientific studies. In addition, the binding interoperability requirements reduce the effort associated with data translation and integration in different systems.

Economic forecasts predict that the EHDS will generate cost savings of € 11 billion in its first ten years of existence. The environmental benefits are also an important component of this economic value proposition. The regulation’s focus on standardized interfaces and data formats creates efficiency gains that translate into both economic and environmental benefits [ 17 ].

The EHDS provides three major pathways to environmental sustainability:

- Cross-Border Efficiency: Standardized international patient summaries (IPS) and electronic prescriptions for primary data usage increase medical efficiency and reduces physical document transport for mobile populations within the European Union;
- Research Infrastructure: Secondary data usage capabilities enable large-scale health research without requiring separate data collection efforts across member states;
- Technical Standardization: Mandatory interoperability requirements based on well-established international standards reduce computational overhead and energy consumption associated with data integration across healthcare systems.

### 4.3 Interoperability Research and Implementation

Foundational research in interoperable health information system architectures provided technical frameworks for environmentally sustainable implementation in digital health. The authors’ earlier work on patient summary generation within large-scale electronic health record networks as a precursor of the EHDS demonstrates how standardized approaches can minimize information redundancy while improving clinical utilization [ 18 ]. Such architectural approaches enable healthcare systems to efficiently share comprehensive patient information and to reduce the need for multiple data collection and storage.

In addition, the authors provided an approach of a technical framework for automated patient summary creation using Clinical Document Architecture and IHE Cross-Enterprise Document Sharing frameworks [ 19 ]. Their research provides guidance for implementing patient summary systems that reduce information overflow for healthcare providers while minimizing computational resources. These efficiency gains lead directly to environmental benefits through reduced energy consumption for data center operations and improved healthcare workflow efficiency.

## 5. Care Collaboration and Virtual Care Delivery

### 5.1 Telemedicine Environmental Impact Mechanisms

Care collaboration and virtual healthcare delivery represent the most direct pathway through which digital health technologies reduce environmental impact. By reducing the need for patient travel to healthcare facilities or support doctors to virtually collaborate, tele-consultations avoid the substantial carbon emissions associated with transportation while often providing comparable or improved clinical outcomes for certain situations.

The environmental benefits of telemedicine are particularly evident in healthcare systems that service geographically dispersed populations. Rural communities often require patients to travel significant distances for specialist consultations, creating substantial transportation-related emissions. Virtual care delivery can reduce this travel necessity while providing access to specialized care that might otherwise be unavailable [ 20 ].

These statements are also underpinned by the authors’ own experiences, who have seen first-hand that patients in Latin America often must travel 1.5 hours by plane to get to the nearest hospital, and then often make a second trip shortly afterwards to discuss their findings.

In summary, it can be said that every trip that patients avoid making to their healthcare facilities reduces the carbon footprint. It is therefore understandable that different medical specialties, which require different numbers of patient contacts, also result in different carbon dioxide emissions. The comparison is shown in Figure 4 . Mental health services and oncological treatments are the strongest drivers here, and the increased use of virtual care technologies generates the greatest benefit in these areas [ 21 ].

**Figure 4:**
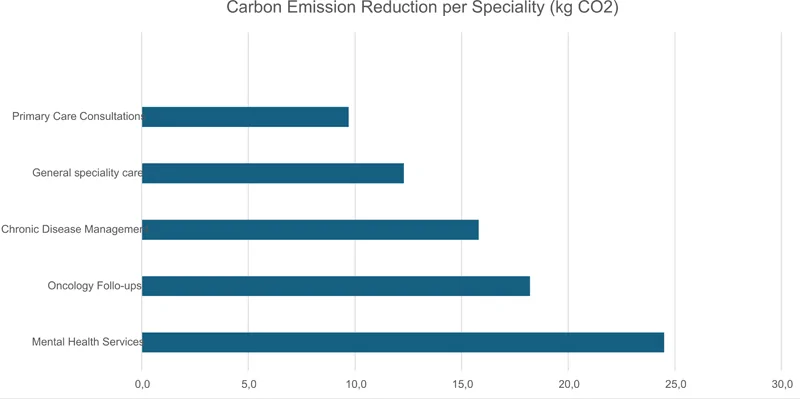
Comparison of carbon emission reductions achieved through virtual care delivery by different medical specialties.

Of course, the question of energy consumption by telemedicine infrastructure also arises. Life cycle analyses consistently show that the energy consumption of such solutions accounts for only a minimal fraction of the environmental benefits achieved by avoiding transport. For instance, comprehensive analyses by Holmner and colleagues have shown that replacing in-person visits with virtual consultations leads to a 40- to 70-fold reduction in CO2 emissions, with the exact magnitude naturally depending on the travel distance, mode of transport, and duration of the consultation [ 22 ].

### 5.2 Specialty-Specific Implementation Patterns

The reductions in carbon emissions achieved through virtual care highly depend on the medical specialty. This reflects the differences in clinical appropriateness and technology adoption rates. While mental health services particularly benefit from virtual care (psychiatric and psychological consultants often rely primarily on verbal communication and observations), it has less impact on physical examinations and diagnostic procedures.

Another area that shows substantial environmental benefits based on virtual care delivery are oncology follow-ups. Cancer patients commonly require frequent monitoring visits that focus on symptom assessment, treatment responses, evaluations and psychological support. Many of these consultations and appointments can be conducted efficiently using video conferencing, which reduces the burden of patients traveling to the providing institution. This is also a major benefit for patients that are already dealing with significant illness-related challenges [ 23 ].

Chronic disease management programs show promising results in combining environmental benefits with even improved clinical outcomes. This is achieved by leveraging remote monitoring technologies for continuous surveillance of vital signs and symptom patterns without frequent in-person visits. This approach shows superior clinical information compared to episodic on-site visits while additionally reducing emissions related to transportation [ 24 ].

### 5.3 Regional Implementation Models and Outcomes

Comparison of virtual care implementation projects in various regions shows different approaches for achieving environmental benefits through the introduction of digital health technologies (see Table 2 ). These approaches show insights into policy strategies, technological choices, and implementation patterns that can optimize clinical and environmental outcomes.

**Table 2:** Comparison of regional approaches to virtual care implementation that show diverse pathways to achieve environmental sustainability goals.

**Region**	Implementation Strategy	Technological Focus	Environmental Outcome
Europe	Regulatory standardization	Interoperable platforms	NHS (UK)
United States	Market-driven innovation	Proprietary platforms	Regional health networks
Latin America	Public and private health networks	University based systems and private provider-based platforms	Reduction of geographic barriers
Asia-Pacific region	Government supported scaling	Mobile first approaches	Population-level implementation

Table 2 : Comparison of regional approaches to virtual care implementation that show diverse pathways to achieve environmental sustainability goals.

#### 5.3.1 United States: Veterans Health Administration Model

In the United States, the U.S. Veterans Health Administration (VA) has developed one of the most comprehensive virtual care programs with the aim of providing instructive lessons for large-scale sustainable healthcare delivery. The VA’s approach focuses on standardization of platforms and protocols to provide virtual care delivery across different geographical regions and clinical specialties. The video platform used provides secure, user-friendly access to healthcare services directly from the patients’ homes. This reduces the need to travel that would generate substantial transportation emissions. By handling over one million annual telehealth encounters addressing geographically dispersed veterans, the VA system demonstrates how institutional commitment supported by a technological standardized infrastructure can achieve substantial environmental benefits and improve the access to care [ 25 ].

#### 5.3.2 Latin America: Network-Based Approaches

In Brazil, the Rede Universitária de Telemedicina (RUTE) provides a model of how university-based networks can achieve environmental benefits while addressing healthcare access challenges. Over the last 16 years, the RUTE connected 140 healthcare facilities across Brazil. They enable specialist consultations, medical education and diagnostic support by removing extensive traveling requirements [ 26 ].

This example showcases how academic medical centers can provide sustainable healthcare delivery networks. Supported by the university’s infrastructure and experience, the system provides high-quality virtual care. Additionally, this is used to train the next generation of healthcare professionals in sustainable practice models. The network’s size and the years of operation provide a good example of network-based approaches to environmentally sustainable healthcare delivery.

#### 5.3.3 Asia-Pacific: Mobile-First Implementation

In the Asia-Pasific region, virtual care approaches focus on mobile platform technologies, which align with regional infrastructure patterns and consumer preferences. The rapid growth of smartphone-based healthcare applications especially in the COVID-19 pandemic shows the potential of mobile-first approaches to achieve population scale environmental benefits [ 27 ].

Countries such as Australia have extended their public healthcare coverage with virtual consultations, which creates a policy framework that supports clinical access and environment sustainability. The integration of virtual care into already existing healthcare systems and processes demonstrates how policy support can accelerate the adoption and growth, while ensuring access to healthcare services in parallel to environmental benefits.

## 6. Patient Engagement and Self-Management Technologies

### 6.1 Digital Tools for Sustainable Health Behavior

PE technologies are an enabler for achieving environmental sustainability and benefits by triggering a digital transformation of the healthcare ecosystem. Compared to telemedicine, which focuses on replacing in-person interactions with virtual substitutes, PE tools and systems aim at reducing the footprint of healthcare from an overall perspective, such as through self-management, preventive care and health education. Different services lead to different level of CO2 reduction.

Self-monitoring applications enable tracking of vital signs, symptoms and medication adherence. This can be done by the patients independently, which allows a reduction of frequencies of routine monitoring visits and shows superior continuous data compared to episodic clinical assessments. These tools have been particularly successful for chronic disease management, where consistency of monitoring together with lifestyle modifications can prevent complications and therefore avoid resource intensive interventions [ 15 ].

Health education platforms are another aspect of reducing healthcare utilization through preventive behavior adaption and improved health literacy. These tools can reach large audiences efficiently, which allows the promotion of behaviors that reduce the risk of diseases and therefore the associated healthcare resource consumptions. The scalability of digital education creates opportunities for population-level environmental benefits through the reduction of healthcare utilization [ 28 ].

### 6.2 Sustainable Technology Design and Implementation

A critical aspect for leveraging the potential of environmental sustainability of PE tools and technologies is an implementation and design approach that considers the complete technology lifecycle. This has been highlighted by recent research focusing on the importance to incorporate sustainable principles already in early developments of digital health tools – including considerations of device longevity, energy consumption patterns and end-of-life disposal strategies [ 21 ].

To achieve this, minimization of electronic waste while maintaining clinical effectiveness is a crucial aspect. Innovative approaches to ensure the sustainability of technology deployment supported by reusable and refurbishment device programs have proven to extend equipment lifespan especially for monitoring devices reducing manufacturing demand and disposal requirements. These circular economy approaches can achieve both environmental and economic benefits while maintaining or improving patient care quality [ 29 ].

### 6.3 Implementation Barriers and Equity Considerations

Independent of the demonstrated and proven environmental benefits, PE tools and platforms with digital health technologies are confronted with implementation challenges that must be addressed to realize its sustainability potential. This especially addresses areas such as digital equity, disparities in technology access, digital literacy and internet connectivity, which can limit clinical benefit and environmental impact. Both aspect populations might benefit most of sustainable delivery models for healthcare.

Especially, health literacy is considered as a barrier to effective patient engagement technology implementation in projects. To efficiently benefit from the possibilities of introduced tools, it is required for users to understand complex health information, navigate sophisticated interfaces and interpret monitoring data accordingly. Failing to address these literacy requirements can render tool utilization ineffective and consequently fail to achieve clinical or environmental benefits [ 28 ].

Another concern that can limit the adoption of PE technology are privacy and security considerations. This particularly affects populations with negative experiences with healthcare systems or technology companies. Building trust through transparency regarding data practices, robust security measures and community engagement are critical success factors for achieving widespread adaptions that lead to population-level environmental benefits.

## 7. Environmental Benefits and Impacts on Healthcare Systems

### 7.1 Quantitative Environmental Impact Analysis

The accumulated evidence from multiple systematic reviews and implementation studies provides compelling quantitative evidence for the environmental benefits of digital health technologies [ 4 ]. The most comprehensive data synthesis comes from systematic reviews that have analyzed dozens of individual studies across diverse healthcare contexts and geographic regions.

#### Quantified Environmental Benefits

- Carbon Emission Reduction: Purohit *et al.* [ 9 ] analyzed 14 studies and found carbon footprint savings ranging from 0,70 to 372 kg CO2 equivalent per virtual consultation. Van der Zee *et al.* [ 10 ] confirmed this range in their updated review with most studies reporting savings between 10 and 50 kg CO2 per consultation;
- Technology Infrastructure Impact: Holmner *et al.* [ 22 ] conducted life cycle assessments showing telemedicine equipment consuming 0,04-0,052 kg CO2 per consultation hour. Additional studies cited by Purohit *et al.* confirmed that technology energy consumption represents less than 1% of the total emission savings achieved [ 9 ];
- Travel Distance Avoidance: Specific travel distance data varies by study context. Studies analyzed by Purohit *et al.* included findings from Robinson and colleagues reporting average savings of 1061 km per consultation in rural Texas settings [ 30 ], while Paquette *et al.* found 50.2 km average distance savings in Michigan [ 31 ]. Systematic reviews indicate median savings typically range from 30 to 100 km depending on the healthcare system structure and density of population [ 9 ];
- Specialty-Specific Variation: Purohit *et al.* identified highest benefits in telepsychiatry and oncology consultations [ 9 ], with studies from Penaskovic *et al.* specifically documenting mental health service benefits [ 23 ]. Primary care and chronic disease management show moderate, but consistent benefits across multiple studies analyzed;
- Geographic Factors: Multiple studies within the reviews show x-fold higher environmental benefits in rural settings compared to urban settings, which reflects longer travel distances to healthcare facilities [ 9 ].

The variation in environmental benefits reflects the connection of multiple factors including the healthcare system structure, transportation, density of population, and technology adoption. It is essential to know and understand these factors for predicting and optimizing environmental outcomes in new implementation contexts.

### 7.2 Synergistic Healthcare and Environmental Benefits

Digital health technologies represent socially relevant value streams that go far beyond direct improvements to the healthcare system and bring broader benefits for the environment. Such synergistic effects strengthen economic efficiency when implemented sustainably. When digital health technologies are used, they create positive feedback loops that can accelerate clinical and environmental benefits.

Virtual healthcare facilitates access to healthcare services for people, especially those in rural or geographically isolated areas [ 32 ]. By overcoming geographical distances, digital health technologies can reduce inequalities in healthcare provision while also benefiting the environment. This alignment of equality and sustainability goals can create opportunities for policy frameworks that address multiple societal challenges simultaneously [ 33 ]. Efficiency improvements through the introduction of digital health technologies lead to economic and environmental improvements. They reduce administrative burdens, improve coordination, and optimize the use of resources. This allows them to reduce costs while reducing environmental impact. This synergy implies incentives for promoting sustainable, environmentally friendly business models [ 32 ].

### 7.3 System-Level Transformation Potential

Digital health transformation can bring about systemic changes that fundamentally alter healthcare structures.

Rather than simply replacing face-to-face interactions with virtual ones, the widespread implementation of digital health can enable new models of care that are inherently more sustainable and effective than traditional/previous approaches.

Preventive models that enable early intervention through health education and screening can reduce the need for resource-intensive acute medical interventions. Through early detection of health problems and self-determined/informed actions, digital health tools can prevent complications that might otherwise require emergency room visits, hospital stays, and intensive treatment with significant environmental impacts [ 34 ].

Approaches to population health management enabled by digital health data analytics can optimize resource allocation and reduce waste across the healthcare system. By analyzing patterns of healthcare utilization or treatment effectiveness and resource use, healthcare organizations can identify opportunities to improve both clinical outcomes and environmental sustainability simultaneously.

## 8. Discussion and Future Directions

### 8.1 Integration of Environmental and Clinical Objectives

The results of this analysis show that environmental sustainability and optimal healthcare are not competing goals but can complement each other.

However, this requires healthcare organizations to broaden their definition of values and include environmental outcomes, particularly carbon footprints, in their decisions and planning alongside traditional clinical and economic metrics [ 35 ].

Quality improvement frameworks that increasingly take sustainability metrics into account and create accountability mechanisms, which support both environmental and clinical goals, can help in this regard. Medical professional organizations are beginning to develop or revise guidelines that consider environmental impacts as part of clinical decision-making [ 35 , 36 ].

### 8.2 Policy Framework Development

The EHDS establishes a comprehensive regulatory framework for digital health implementation, covering both primary and secondary use of health data across EU member states [ 16 ], with implementation extending through 2030 [ 37 ].

With its focus on standardization, interoperability, and data exchange, it creates a model that other regions of the world could adapt to their specific circumstances and healthcare structures. The EHDS shows how regulatory frameworks can simultaneously ensure clinical quality, the protection of sensitive patient data, the promotion of innovation, and environmental sustainability.

Successful implementation also requires careful consideration of gender equality aspects. The digital divide can hinder acceptance and therefore remains a major challenge if not addressed through targeted measures. Policy frameworks must ensure equitable access to digital health technologies and the knowledge necessary for their effective use [ 38 ].

### 8.3 Research Priorities and Methodological Needs

The systematic application of the search strategy and the identification of relevant literature assisted by Claude.ai proved to be efficient.

However, there is an urgent need to develop standardized methodological frameworks for environmental impact assessment. Current studies use different approaches, which makes comparative analyses difficult and limits the predictability of ecological outcomes in new contexts [ 11 ].

Longitudinal studies are needed to examine the sustainable environmental impacts of the introduction of digital health technologies to understand how the benefits evolve over time and to which degrees. Current studies focus on short-term impacts in the initial implementation phase, but the long-term impacts of the systematic, widespread introduction of digital health technologies are still largely unknown.

In addition, research is needed to investigate the environmental impacts of new technologies such as artificial intelligence and Internet of Things devices, as these technologies are increasingly used in healthcare applications. Understanding how to optimize the environmental benefits of these highly computationally intensive technologies will be critical to maintaining favorable environmental performance.

### 8.4 Implementation Challenges and Solutions

The successful implementation of environmentally sustainable digital health technologies requires overcoming numerous challenges in technical, organizational, and social areas. The lack of interoperability between technologies remains a significant barrier in many health information systems and limits the efficiency gains that could be achieved through seamless data exchange and coordinated care.

Managing the accompanying organizational changes is another critical factor for successful implementation. Healthcare providers must develop new workflows, skills, and performance indicators that encompass both clinical excellence and environmental sustainability. This transformation requires systemic leadership, talented employees, and a cultural change that can take many years to fully implement.

The acceptance and use of digital health technologies by patients can vary significantly depending on the population group and clinical context. Successful implementation requires the understanding and consideration of various factors such as age, health literacy, familiarity with technologies, and cultural preferences. Tailored approaches are essential to achieve broad acceptance.

## 9. Conclusions

This comprehensive analysis shows evidence that digital health technologies offer substantial opportunities for reducing healthcare’s environmental impact while maintaining or improving clinical outcomes. The consistency of findings over multiple systematic reviews, healthcare contexts and different geographic regions supports confident impression about the environmental benefits of digital health information systems.

The aim of this work was to analyze whether and to what extent HIE, CC, and PE can contribute to reducing the share of the carbon footprint caused by healthcare. The three domains that have been examined complementary contribute to achieving environmental sustainability. HIE reduces paper consumption, conventional postal service and redundancies of tests and examinations. CC directly reduces transportation-related emissions while improving healthcare access. PE technologies reduce overall healthcare utilization through improved self-management, improved healthcare access, and better preventive care.

The quantitative evidence is particularly compelling, with carbon emission reductions ranging from 0.70 to 372 kg of CO2 equivalent per virtual consultation and technology infrastructure representing less than 1% of total environmental benefits achieved. This ratio creates strong environmental value propositions that strongly go in line with health-related and economic goals.

Regional implementation examples from Europe, North America, Latin America, and Asia-Pacific suggest that environmental benefits can be achieved through diverse policy approaches and technological strategies. The EHDS in Europe provides, among other benefits, a comprehensive framework for integrating environmental sustainability into digital health strategies, while other regions offer important lessons about market-driven innovation like in the United States, network-based collaboration in Latin America, and some mobile-first implementation approaches in the Asia-Pacific region.

However, realizing the full environmental potential of digital health technologies requires addressing significant implementation challenges. Furthermore, digital equity concerns must be addressed to ensure that benefits reach all populations to ensure the highest possible level of acceptance and dissemination, which in turn is a prerequisite for achieving environmental benefits. Methodological standardization is needed to improve environmental impact assessment and planning.

Looking ahead, it would be very helpful if standardized evaluation and measurement methods were developed that would also assess and compare the environmental impact, particularly CO2 emissions, of digital transformation systems when they are introduced—not only for use in health information systems but also in other areas. The development and introduction of such systems require interdisciplinary collaboration between healthcare providers, environmental scientists, technology developers, and policymakers to maximize both clinical and environmental benefits.

As healthcare systems around the world face the dual challenge of improving population health and combating climate change, digital health technologies are becoming indispensable tools [ 38 ].

The evidence presented clearly supports accelerated strategic investment in the rapid expansion of sustainable digital health solutions that protect both human health and the health of our planet for current and future generations. These facts should be considered by decision-makers in their investment decisions.

## 10. Acknowledgment and Conflicts of Interest

The authors possess expertise in medicine, information technology, and economics, supported by an extensive academic track record. In their primary roles at Siemens Healthineers, they develop and operate health technology, engage in multiple research projects, and actively contribute to scientific communities. This article was prepared on their own initiative in their personal time. Combating climate change is a cause to which they are deeply committed. The authors declare no conflicts of interest and report receiving no funding for this work from Siemens Healthineers or from any other source.

## Footnotes

1 Claude AI was used to assist with preliminary title / abstract screening and aggregation of eligible publications.

## References

[1] Watts N, Amann M, Arnell N, Ayeb-Karlsson S, Beagley J, Belesova K, *et al.* The 2020 report of The Lancet Countdown on health and climate change: responding to converging crises. The Lancet 2021;397:129–70. https://doi.org/10.1016/S0140-6736(20)32290-X 10.1016/S0140-6736(20)32290-X PMC7616803 33278353

[2] Keil M, Frehse L, Hagemeister M, Knieß M, Lange O, Kronenberg T, *et al.* Carbon footprint of healthcare systems: a systematic review of evidence and methods. BMJ Open 2024;14:e078464. https://doi.org/10.1136/bmjopen-2023-078464 10.1136/bmjopen-2023-078464 PMC11086491 38688670

[3] Gaetani M, Uleryk E, Halgren C, Maratta C. The carbon footprint of critical care: a systematic review. Intensive Care Med 2024;50:731–45.https://doi.org/10.1007/s00134-023-07307-1 10.1007/s00134-023-07307-1 38416200

[4] Rodríguez‐Jiménez L, Romero‐Martín M, Spruell T, Steley Z, Gómez‐Salgado J. The carbon footprint of healthcare settings: A systematic review. J Adv Nurs 2023;79:2830–44.https://doi.org/10.1111/jan.15671 10.1111/jan.15671 37198974

[5] Rizmie D. Digital Health Innovations Tackling the Climate Crisis | Center for Global Digital Health Innovation. Cent Glob Digit Health Innov John Hopkins Bloom Public Sch Health 2024.https://publichealth.jhu.edu/center-for-global-digital-health-innovation/june-2024-digital-health-innovations-tackling-the-climate-crisis(accessed August 20, 2025)

[6] Aboye GT, Vande Walle M, Simegn GL, Aerts J-M. mHealth in sub-Saharan Africa and Europe: A systematic review comparing the use and availability of mHealth approaches in sub-Saharan Africa and Europe. Digit Health 2023;9:20552076231180972.https://doi.org/10.1177/20552076231180972 10.1177/20552076231180972 PMC10291558 37377558

[7] Bloomfield P, Clutton-Brock P, Pencheon E, Magnusson J, Karpathakis K. Artificial Intelligence in the NHS: Climate and Emissions*,**. J Clim Change Health 2021;4:100056.https://doi.org/10.1016/j.joclim.2021.100056

[8] Rahimi-Ardabili H, Magrabi F, Coiera E. Digital health for climate change mitigation and response: a scoping review. J Am Med Inform Assoc 2022;29:2140–52.https://doi.org/10.1093/jamia/ocac134 10.1093/jamia/ocac134 PMC9667157 35960171

[9] Purohit A, Smith J, Hibble A. Does telemedicine reduce the carbon footprint of healthcare? A systematic review. Future Healthc J 2021;8:e85–91.https://doi.org/10.7861/fhj.2020-0080 10.7861/fhj.2020-0080 PMC8004323 33791483

[10] Van Der Zee C, Chang-Wolf J, Koopmanschap MA, Van Leeuwen R, Wisse RP. Assessing the Carbon Footprint of Telemedicine: A Systematic Review. Health Serv Insights 2024;17:11786329241271562.https://doi.org/10.1177/11786329241271562 10.1177/11786329241271562 PMC11320403 39139395

[11] Lokmic-Tomkins Z, Davies S, Block LJ, Cochrane L, Dorin A, Von Gerich H, *et al.* Assessing the car-bon footprint of digital health interventions: a scoping review. J Am Med Inform Assoc 2022;29:2128–39. https://doi.org/10.1093/jamia/ocac196 10.1093/jamia/ocac196 PMC9667173 36314391

[12] Braithwaite J, Smith CL, Leask E, Wijekulasuriya S, Brooke-Cowden K, Fisher G, *et al.* Strategies and tactics to reduce the impact of healthcare on climate change: systematic review. BMJ 2024:e081284. https://doi.org/10.1136/bmj-2024-081284 10.1136/bmj-2024-081284 PMC11459334 39379104

[13] Faculty of Computing and Software Engineering, Arba Minch University, Arba Minch, Ethiopia, Arega A, Sharma DP. Evaluating Energy-efficiency and Performance of Cloud-based Healthcare Systems Using Power-aware Algorithms: An Experimental Simulation Approach for Public Hospitals. Int J Inf Technol Comput Sci 2025;17:72–96.https://doi.org/10.5815/ijitcs.2025.03.06

[14] Guo Y, Ganti S, Wu Y. Enhancing Energy Efficiency in Telehealth Internet of Things Systems Through Fog and Cloud Computing Integration: Simulation Study. JMIR Biomed Eng 2024;9:e50175.https://doi.org/10.2196/50175 10.2196/50175 PMC11041449 38875671

[15] Greenwood DA, Gee PM, Fatkin KJ, Peeples M. A Systematic Review of Reviews Evaluating Tech-nology-Enabled Diabetes Self-Management Education and Support. J Diabetes Sci Technol 2017;11:1015–27.https://doi.org/10.1177/1932296817713506 10.1177/1932296817713506 PMC5951000 28560898

[16] European Health Data Space Regulation (EHDS). Eur Comm 2025.https://health.ec.europa.eu/ehealth-digital-health-and-care/european-health-data-space-regulation-ehds_en(accessed August 20, 2025)

[17] Marcus JS, Martens B, Carugati C, Bucher A, Godlovitch I. The European Health Data Space. SSRN Electron J 2022.https://doi.org/10.2139/ssrn.4300393

[18] Mangesius P, Saboor S, Schabetsberger T. Patient Summaries in Context of Large Scale EHR Net-works with Fine Granular Access Control Restrictions. Stud Health Technol Inform 2017;237:91–6 28479549

[19] Mangesius P, Saboor S, Healy T, Schabetsberger T. Dynamic Creation of Patient Summaries: A CDA and IHE XDS Based Approach for Regional EHRs. Stud Health Technol Inform 2017;245:170–3 29295075

[20] Lathan R, Hitchman L, Walshaw J, Ravindhran B, Carradice D, Smith G, *et al.* Telemedicine for sustainable postoperative follow-up: a prospective pilot study evaluating the hybrid life-cycle as-sessment approach to carbon footprint analysis. Front Surg 2024;11:1300625. https://doi.org/10.3389/fsurg.2024.1300625 10.3389/fsurg.2024.1300625 PMC10982357 38562585

[21] Thiel CL, Mehta N, Sejo CS, Qureshi L, Moyer M, Valentino V, *et al.* Telemedicine and the envi-ronment: life cycle environmental emissions from in-person and virtual clinic visits. Npj Digit Med 2023;6:87. https://doi.org/10.1038/s41746-023-00818-7 10.1038/s41746-023-00818-7 PMC10169113 37160996

[22] Holmner Å, Ebi KL, Lazuardi L, Nilsson M. Carbon Footprint of Telemedicine Solutions - Unexplored Opportunity for Reducing Carbon Emissions in the Health Sector. PLoS ONE 2014;9:e105040.https://doi.org/10.1371/journal.pone.0105040 10.1371/journal.pone.0105040 PMC4154849 25188322

[23] Penaskovic KM, Goldenberg MN, Gerkin JS. Telepsychiatry: a Potential Force Against Climate Change. Acad Psychiatry 2021;45:403–4.https://doi.org/10.1007/s40596-021-01452-9 10.1007/s40596-021-01452-9 PMC8032316 33834420

[24] Brownson CA, Hoerger TJ, Fisher EB, Kilpatrick KE. Cost-effectiveness of Diabetes Self-management Programs in Community Primary Care Settings. Diabetes Educ 2009;35:761–9.https://doi.org/10.1177/0145721709340931 10.1177/0145721709340931 19622716

[25] Heyworth L, Shah N, Galpin K. 20 Years of Telehealth in the Veterans Health Administration: Taking Stock of Our Past and Charting Our Future. J Gen Intern Med 2024;39:5–8.https://doi.org/10.1007/s11606-024-08617-w 10.1007/s11606-024-08617-w PMC10937874 38378981

[26] Soriano Marcolino M, Minelli Figueira R, Pereira Afonso Dos Santos J, Silva Cardoso C, Luiz Ribeiro A, Alkmim MB. The Experience of a Sustainable Large Scale Brazilian Telehealth Network. Telemed E-Health 2016;22:899–908.https://doi.org/10.1089/tmj.2015.0234 10.1089/tmj.2015.0234 27167901

[27] Kapur V, Boulton A. Covid-19 Accelerates the Adoption of Telemedicine in Asia-Pacific Countries. Bain 2020.https://www.bain.com/insights/covid-19-accelerates-the-adoption-of-telemedicine-in-asia-pacific-countries/(accessed August 20, 2025)

[28] Norman CD, Skinner HA. eHealth Literacy: Essential Skills for Consumer Health in a Networked World. J Med Internet Res 2006;8:e9.https://doi.org/10.2196/jmir.8.2.e9 10.2196/jmir.8.2.e9 PMC1550701 16867972

[29] Hameed K, Naha R, Hameed F. Digital transformation for sustainable health and well-being: a review and future research directions. Discov Sustain 2024;5:104.https://doi.org/10.1007/s43621-024-00273-8

[30] Robinson JD, Prochaska JD, Yngve DA. Pre-surgery evaluations by telephone decrease travel and cost for families of children with cerebral palsy. SAGE Open Med 2017;5:2050312117720046.https://doi.org/10.1177/2050312117720046 10.1177/2050312117720046 PMC5528192 28839936

[31] Paquette S, Lin JC. Outpatient Telemedicine Program in Vascular Surgery Reduces Patient Travel Time, Cost, and Environmental Pollutant Emissions. Ann Vasc Surg 2019;59:167–72.https://doi.org/10.1016/j.avsg.2019.01.021 10.1016/j.avsg.2019.01.021 31077768

[32] Pearl R. Kaiser Permanente Northern California: Current Experiences With Internet, Mobile, And Video Technologies. Health Aff (Millwood) 2014;33:251–7.https://doi.org/10.1377/hlthaff.2013.1005 10.1377/hlthaff.2013.1005 24493768

[33] Reed ME, Huang J, Graetz I, Lee C, Muelly E, Kennedy C, *et al.* Patient Characteristics Associated With Choosing a Telemedicine Visit vs Office Visit With the Same Primary Care Clinicians. JAMA Netw Open 2020;3:e205873. https://doi.org/10.1001/jamanetworkopen.2020.5873 10.1001/jamanetworkopen.2020.5873 PMC7301227 32585018

[34] Schwamm LH, Erskine A, Licurse A. A digital embrace to blunt the curve of COVID19 pandemic. Npj Digit Med 2020;3:64.https://doi.org/10.1038/s41746-020-0279-6 10.1038/s41746-020-0279-6 PMC7198549 32377575

[35] Bangert MLN Fahmida. Health care innovations in sustainability will protect both patients and the planet. Trellis 2024.https://trellis.net/article/health-care-innovations-sustainability-will-protect-both-patients-and-planet/(accessed August 20, 2025)

[36] Knagg R, Dorey J, Evans R, Hitchman J. Sustainability in healthcare: patient and public perspectives. Anaesthesia 2024;79:278–83.https://doi.org/10.1111/anae.16159 10.1111/anae.16159 38205532

[37] Quathem KV. EHDS Series – 5: European Health Data Space Governance, Enforcement and Time-lines. Covington Digit Health 2024.https://www.covingtondigitalhealth.com/2024/04/ehds-series-5-european-health-data-space-governance-enforcement-and-timelines/(accessed August 21, 2025)

[38] Global Strategy on Digital Health 2020-2025. 1st ed. Geneva: World Health Organization; 2021.https://www.who.int/docs/default-source/documents/gs4dhdaa2a9f352b0445bafbc79ca799dce4d.pdf(accessed August 20, 2025)

